# Case of Induction of Labor at the Seventh Month, by the Water Douche

**Published:** 1855-09

**Authors:** Andrew Cheeseman


*Case of Induction of Labor at the Seventh Month, by the Water Douche.* By ANDREW CHEESEMAN, M. D.

In the month of November, 1853, I attended Mrs. L., residing in Kates street, in her first confinement. There was contraction at the inferior strait; the transverse diameter being 2 inches. After a severe labor of 48 hours the head was perforated and with considerable difficulty a large child was extracted. Vaginitis, supervened and her recovery was tedious. Being informed of her unfortunate condition, she promised if again pregnant, to give me early intimation of the fact; and accordingly in June last told me that her last menstrual period terminated on the 5th of January, and she had quickened in the last week in April. With her consent I determined to induce premature labor and to adopt the method of Prof. Kiwisch, of Wurtzburg.

August 10th, 4 o'clock, P. M.—Touched and found the os high up and undilated. Placing a bucket about four feet above the bed on which the patient lay, by means of a few feet of india rubber tubing, terminating in a nozzle, and used as a syphon, I directed a small stream of tepid water on the os uteri for 20 minutes, using in that time six gallons of water. She complained of no pain or uneasiness, and on examination I found no perceptible alteration in the parts.

August 11th, 9 o'clock, A. M.—I found, to my surprise, that my patient was in labor, the pains having commenced at 7 o'clock this morning. On touching I found the os quite low down an inch and a half in diameter, and the membranes protruding strongly. Repeated the douche for 15 minutes.

11 o'olock, A. M.—Os uteri fully dilated. Membranes filling the vagina and reaching the vulva.

12 o'clock, M.—Membranes ruptured. Feet presenting with toes to the pubis and the cord prolapsed. The body was slowly delivered without difficulty, and the right arm and shoulder came out from under the pubic arch; but the left shoulder and arm caught behind a strong transverse band (a result, doubtless, of the vaginitis following the previous labor,) which extended from side to side of the pelvis across the lower part of the sacrum, firm and tense, with a sharp crescentic edge and fully three quarters of an inch broad at its middle. It was with some difficulty that I carried the arm over the face and brought it down, during which operation the child gasped strongly twice. The mouth and afterwards the nose caught on the same obstruction, and I was compelled to use the rectis in order to relieve and extract the head. The child was still born. The placenta followed in two or three minutes.

The chief points of interest in this case are the ease and promptness with which labor was induced, and the obstruction that impeded its progress. In all the cases I have seen noticed in the journals, the number of applications of the douche, and the time required to bring on the labor, was much greater than in this case; when the process commenced in 15 hours and terminated in 20 hours after the first application of the douche, and proceeded so far before the second application that it is doubtful whether more than the first was necessary. The unfortunate presence of the obstructing band, and the presentation of the feet, frustrated the chief end for which the labor was prematurely induced. Had the head presented, or with the feet presenting had there been no unusual impediment, I feel assured that the child would have survived the labor.

[The injection of water into the uterus, as a means of producing premature delivery, has been known, we have reason to believe, in this country for some time, though no cases that we are aware of have been recorded illustrative of its use. In a work, however, on Materia Medica, by Dr. J. B. Biddle, of this city, published in January, 1852, we find among the uses of *cold water* the following:—"Cold water, injected into the impregnated uterus, is among the most certain means of inducing premature delivery."—Ed. Ex.]

